# The right dose of data: balancing reliability and participant burden in a validated daily measure of food parenting practices

**DOI:** 10.1186/s12966-025-01855-z

**Published:** 2025-11-26

**Authors:** Olivia De-Jongh González, Louise C. Mâsse

**Affiliations:** 1 School of Population and Public Health, University of British Columbia, BC Children’s Hospital Research Institute, F609-4480 Oak Street, Vancouver, BC V5Z 4H4 Canada; 2 School of Population and Public Health, University of British Columbia, BC Children’s Hospital Research Institute, F605B-4490 Oak Street, Vancouver, BC V5Z 4H4 Canada

**Keywords:** Food parenting practices, Intensive longitudinal methods, Daily diary, Reliability, Validity, Measurement, Mothers, Fathers

## Abstract

**Background:**

Intensive longitudinal methods are increasingly used to capture daily fluctuations in food parenting practices (FPP), but reliable tools for daily assessment are limited. Most studies rely on a fixed recall period for all FPP constructs; yet, the reliability of such approach remains unclear as some FPP may fluctuate more than others. Identifying the minimum number of measurement days needed can improve the feasibility and quality of daily FPP research. This Daily Diary study aimed to: 1) assess the test–retest reliability of daily FPP measures among mothers and fathers over a 14-day period; 2) estimate the minimum number of days needed to achieve adequate reliability; and 3) validate these reliability estimates using a second 14-day period.

**Methods:**

A total of 315 parents (aged 40 ± 5 years; 60% mothers) of children aged 2.5–6 years completed 28 daily assessments across two 14-day periods, three months apart, yielding 8820 observations. Seven FPP constructs were assessed daily using 13 binary items, and evidence of validity was obtained with a Confirmatory Factor Analysis (CFA). Intraclass correlation coefficients (ICCs) were calculated from mixed-effect models using data from the first 14-day period. The Spearman-Brown formula estimated the minimum number of days needed for ICC ≥ 0.70. These minimum-day estimates were validated using the second 14-day period, drawing random sets of consecutive and non-consecutive days.

**Results:**

ICCs in the first period ranged from 0.80–0.97. Required days for acceptable reliability varied by FPP construct: Coercive control (1 day); Autonomy support, Child involvement, and Meal routines (5–6 days); and Rules and limits, Nondirective support, and Healthy opportunities (8–9 days). Compared to fathers, mothers required fewer days to reliably assess Rules and Limits, Healthy Opportunities, Autonomy and Nondirective Support. Validation in the second period showed ICCs ≥ 0.70 except for Healthy opportunities, Nondirective support, and Child involvement, which had borderline ICCs (0.63–0.68) in some subgroups and sampling approaches.

**Conclusions:**

This study provides a valid and reliable instrument to measure FPP in Daily Diary protocols. Although 14 days of data collection ensures high reliability, many FPP constructs can be assessed reliably with fewer days. To reduce participant burden, researchers may tailor diary duration based on the specific construct and caregiver sex.

**Supplementary Information:**

The online version contains supplementary material available at 10.1186/s12966-025-01855-z.

## Background

Parents influence children’s eating habits through their food parenting practices (FPP), defined as goal-directed strategies used to regulate children’s dietary behaviors [1]. FPP are commonly organized into three domains [1]. *Controlling* practices involve pressure, restriction and intrusiveness (e.g., pressuring a child to finish their meal without considering hunger/satiety levels, offering foods as reward for good behavior). *Structured* practices consist of providing consistency and organization in the eating environment through routines, limits, modeling, and exposure to healthy foods (e.g., regular family meals, limits on treats, availability of healthy foods at home). *Autonomy-promoting* practices encourage children’s independence by supporting choice, internal motivation and involvement (e.g., involving children in food decisions and preparation, encouraging them to try new foods).

While extensive research has examined FPP, studies have traditionally treated them as static behaviors by measuring them as “usual” practices, rather than accounting for their daily variations [2]. However, FPP are likely transient and context-dependent, meaning that they could change from one day to another or across different situations, depending on a variety of factors including parent and child mood, stress level, timing of scheduled activities, and child eating behaviors [3–7]. For example, parents may ease up on structured practices during meals when their child is cranky, overtired, or persistently asking for a treat [3], or use controlling practices on more stressful days [6].

In recent years, researchers have increasingly recognized the importance of capturing fluctuations in FPP (i.e., how these practices change from one day to another or within the same day). This has led to a growing number of studies employing intensive longitudinal methods such as Daily Diaries, Experience Sampling, and Ecological Momentary Assessments (EMA) [2, 5–13]. These approaches involve repeated, sequential measurements over short time intervals (e.g., daily, or multiple times per day) to track how emotions and behaviors vary over time [14]. Compared to traditional retrospective assessments that typically ask caregivers to recall their usual FPP over weeks or even months, intensive longitudinal methods reduce recall bias and better capture the dynamic, context-dependent nature of parenting behaviors.

Among these methods, Daily Diaries, typically involving once-per-day assessments, are particularly well-suited for capturing discrete behaviors that may vary from day to day but are still relatively easy for participants to recall [15]. This applies to FPP, which, unlike more subjective experiences (e.g., emotions, thoughts) that fluctuate quickly and are hard to recall accurately, can be captured with less intensive assessments. In fact, Daily Diary designs have been recognized for striking an optimal balance between overly sparse and overly burdensome data collection schedules [15].

Despite the growing use of intensive longitudinal methods to evaluate FPP, this research area is still emerging, with few reliable instruments specifically designed for daily FPP assessment. Moreover, studies that have designed or adapted FPP instruments for intensive assessments rarely report how reliability was evaluated [2, 6–8, 12, 16]. Typical reliability metrics such as Cronbach's alpha evaluate internal consistency (correlation between items) at a single time point, but do not measure stability and consistency over time [17, 18]. However, since evidence shows that FPP are transient and context-dependent [3–7], there is a need for reliability assessments that accurately capture “true” fluctuations in parenting behaviors.

Additionally, existing studies use fixed 5–10-day assessment periods for all FPP constructs [2, 5–10, 13]. However, using the same number of assessments for all FPP constructs may present two key challenges: a) too few assessments may not provide reliable estimates, or b) excessively long assessment periods may impose unnecessary participant burden and research costs if fewer days are sufficient. Furthermore, some FPP may be more stable and as such would require fewer assessments, while others may fluctuate more and require longer assessment periods. Similarly, as caregivers may have different stressor levels, engage in different child-rearing responsibilities and practices, or have different relationships with their child around food [7, 19], their use of FPP may vary, likely requiring assessment periods that can accommodate this level of variability. Therefore, identifying the optimal number of daily assessments to achieve acceptable reliability for each FPP and caregiver type would benefit both researchers and participants, balancing psychometric rigor with feasibility.

To address these gaps, this study had three primary objectives. First, we evaluated the test–retest reliability of FPP measures reported daily by mothers and fathers over a 14-day period. Second, we aimed to determine the minimum number of measurement days required to achieve acceptable reliability, defined as an intraclass correlation coefficient (ICC) ≥ 0.70. Third, we sought to validate these reliability estimates by applying the identified minimum number of days within a second, independent 14-day assessment period, using both randomly selected non-consecutive days and consecutive days.

## Methods

### Design

This study was approved by the Research Ethics Board of the University of British Columbia (H18-01434) as part of the *Good Start Matters* (GSM) *Parenting Project,* a broader study focused on supporting caregivers’ parenting practices related to child diet and active play. We employed a Daily Diary design, an intensive longitudinal method involving daily assessments of FPP across two 14-day periods spaced three months apart. This design was selected to capture FPP as they naturally occur and fluctuate from day to day, thereby enhancing ecological validity, minimizing recall bias, and allowing for the examination of within-person processes over time. Participants received $20 CAD for completing an initial general survey where demographics were measured, and up to $42 CAD for the Daily Diary assessment ($1.50 CAD per completed daily survey).

### Recruitment and data collection

Parents were recruited through their child’s childcare facility in British Columbia, Canada, from November 2023 to January 2025. Data collection took place from November 2023 to April 2025, but it was paused during key holiday periods that may affect food-related practices and routines (Dec 23rd to January 2nd). Recruitment occurred during visits to each childcare center, with most parents enrolling within 1–2 months of the visit, though enrollment remained open as long as the study was actively recruiting. Parents provided either written informed consent by signing printed forms or electronic consent via REDCap by scanning a QR code from posters or accessing a link distributed by email. After consenting, parents received a link to start assessments, beginning with a general survey (day 0) followed by the Daily Diary (days 1–14). Parents were informed that they would receive a daily “check-in” text message with a link to complete a short survey which would take no more than five minutes and expire at midnight each day, and that a second daily check-in period would start approximately three months later. Recruitment and data collection occurred without additional interruptions, ensuring that seasonality effects were similarly distributed across individuals, as assessments took place within approximately a three-month window.

### Participants

Parents were eligible to participate if they had primary or shared custody of a 2.5–6-year-old child, were able to read and understand English, and had a smartphone device to receive daily text messages. This age range was selected to balance developmental relevance, ecological validity, and the logistical constraints of the study. Research indicates that children’s eating behaviors and parental feeding practices are relatively stable between ages 2–5 [20], allowing continuity in FPP measures. Additionally, this study was conducted alongside another study that assessed children’s fundamental movement skills, which would be challenging to evaluate with children under 2.5 years. The upper limit selected (6 years) reflects the recruitment context in childcare settings, where some children are slightly older than five but share similar routines, developmental characteristics, and caregiving environments. Additional eligibility criteria included not being participating in a pediatric weight management or nutritional program or having severe dietary or physical restrictions that would prevent adherence to Canadian dietary and physical activity guidelines. A total of 315 parents participated in this study, which resulted in an analytical dataset of 8820 observations (315 × 14 × 2). The sample size was determined based on the broader GSM project and was not specifically designed for this Daily Diary study.

### Measures

To evaluate FPP, we used 3 items created for this study and 11 items modeled after the validated FPP item bank [21]. The item bank’s conceptual development was informed by a concept mapping analysis of parenting and nutrition experts [22], and its psychometric properties were assessed with both factor analysis and item-response theory, exhibiting adequate reliability and strong construct validity invariant by parental sex [21]. To develop our Daily Diary protocol, we selected a brief set of items relevant to this age range, aiming to assess key FPP constructs while ensuring the tool remained manageable over a 28-day period. The original item bank examines parents’ overall frequency of FPP from never to always on a 1–5-point scale. For our study, the questions were adapted for younger children and modified as binary indicators, evaluating which practices were used each day. Specifically, parents received a daily text message early in the morning, with a link to complete a short survey in REDCap. FPP items asked “*Yesterday I…* [did x]”, with yes/no response options (e.g., “*Yesterday I took away dessert because* [child name] *misbehaved or did not eat all the food I served*”). Supplemental Table S1 shows the full list of items used in this instrument. Seven FPP constructs covering the Control, Autonomy promotion, and Structure parenting domains were evaluated. Control included Coercive control (2 items), Autonomy promotion included Autonomy support (2 items), and Child involvement (2 items), and Structure included Meal routines (2 items), Rules and limits (2 items initially, but 1 item was dropped post Confirmatory Factor Analysis (CFA) as described below), Healthy opportunities (3 items), and Nondirective support (1 item). The Daily Diary survey, which included additional questions not related to the present study, took 3–5 min to complete on average.

### Analysis

Prior to creating daily FPP constructs, missing data (~ 35%) in the binary items were imputed using multivariate imputation by chained equations with a logistic model, generating 20 multiply imputed datasets that were subsequently used in analyses. No participants were excluded. Sensitivity analyses demonstrated that results were consistent when using complete cases, with ICCs across the 14-day period ranging from 0.80–0.97 with imputation and 0.86–0.98 without imputation. Because the imputed datasets allowed us to retain the full sample and conduct day-selection reliability analyses, we decided to report findings from the imputed datasets.

Linear, logistic, and ordered logistic mixed-effects models and their postestimation *estat icc* Stata’s command were used to calculate ICCs for numeric, binary, and ordered FPP constructs, respectively. In logistic models, Stata computes ICC using the latent variable formulation, where the residual variance is fixed at π^2^/3, ensuring comparability with ICC values from linear models [17, 23, 24].

To estimate the number of assessment days needed while balancing psychometric rigor with feasibility, we selected a reliability cutoff of ≥ 0.70. Although coefficients in the 0.80–0.90 range are desirable, a 0.70 threshold is commonly regarded as acceptable [25–27]. To estimate the minimum number of days required to reach this level of reliability, we use the Spearman-Brown prophecy formula, which expresses the reliability of a scale as a function of the reliability of a single measurement [17, 28]. Using the single-day ICC (i.e., ICCi) calculated over the initial 14-day assessment period, we computed the number of days needed to achieve a reliability ≥ 0.70 as follows: *days needed* = *(0.70 (1-ICCi))/(ICCi (1–0.70))*. The required number of days was rounded up in all cases in which decimals were obtained.

We then leveraged the second 14-day assessment period to validate these estimates using the same mixed-effect models and two selection approaches: 1) consecutive days, where a random number determined the starting day for a continuous n-day period with the constraint that the number of days selected could not exceed 14, and 2) random non-consecutive days, where n days were selected randomly from a list of 14 days without the constrain of being consecutive. The number of days selected for each construct was based on the estimates of the minimum number of days required for reliable measurement. While consecutive days reflect how FPP are typically assessed in intensive longitudinal studies, non-consecutive selection allowed us to examine the robustness of the reliability estimates. As participants did not start their 14-day period on the same day of the week or in the same month, potential weekday-weekend effects as well as seasonality influences were already distributed across individuals. However, clustering assessments within a short period (method 1, consecutive days) could still capture reliability differently than more dispersed sampling (method 2, non-consecutive random days). Comparing both methods ensures that findings are robust to different data collection strategies and helps determine whether a more flexible (and potentially less burdensome), non-consecutive approach provides similarly reliable estimates. All analyses, performed in Stata [29], were initially conducted in the full sample and further stratified by parent sex.

While this paper focused on reliability, we also confirmed the instrument’s validity using CFA. The hypothesized seven-factor structure was supported with excellent model fit: χ^2^(46) = 75.06, p = 0.004; Root Mean Squared Error of Approximation (RMSEA) = 0.045 [0.025, 0.063]; Comparative Fit Index (CFI) = 0.94; Tucker-Lewis index (TLI) = 0.97; Standardized Root Mean Squared Residual (SRMR) = 0.04. Factor loadings per item are provided in Supplemental Table S2. One item was dropped (see Table S2), leaving the construct Rules and limits represented by a single item. Single-indicator constructs (Rules and limits, and Nondirective support) were retained in the CFA model to preserve their correlations with other constructs. However, they were considered ‘observed’ rather than ‘latent’ constructs and as such, their factor loadings were fixed at 1 and error variances were set to 0.

## Results

Table 1 presents demographic characteristics at the time of recruitment, as well as descriptive statistics for FPP over the first 14-day period. Overall, we obtained similar distributions by parent and child sex, but a predominance of married or common-law parents, who generally had higher education and income levels. Only three variables significantly differed by parent sex: mothers were, on average, one year younger than fathers (39 versus 41 years, *p* = 0.004), more likely to have a university degree (87% versus 72%, *p* = 0.002), and more likely to involve their child in simple meal tasks (mean frequency 3.27 versus 2.57, *p* = 0.015). Additionally, at baseline, 51% and 9% of parents reported no use (at all) of Coercive control and Child involvement, respectively, while the remaining FPP were used at least once over the 14-day period by 99–100% of parents, with no differences by parent sex.

**Table 1:** Descriptive statistics for demographics at recruitment time and FPP during the first 14-day period

Variables		N or Mean	% or ± SD
Child sex	Male	156	49.52%
	Female	159	50.48%
Child age		4.20	± 0.74
Parent sex	Male	127	40.32%
	Female	188	59.68%
Parent age^a^		39.77	± 5.10
Parent marital status	Married or common-law	284	90.16%
	Single, divorced, or widowed	29	9.21%
Parent education^a^	University degree	254	80.63%
	Non-university degree	60	19.05%
Parent ethnic background	Europe-Canada and -US descent	123	39.05%
	East and Southeast Asian descent	125	39.68%
	South and Southwest Asian descent	39	12.38%
	Latin America descent	19	6.03%
	Other (e.g., First Nations or African descent)	9	2.86%
Household income ($ CAD)	< $50,000	19	6.03%
	$50,000—$99,999	52	16.51%
	$100,000—$149,999	83	26.35%
	> $150,000	139	44.13%
Food Parenting Practices (FPP)	Control domain:		
	Coercive control	1.37	± 2.17
	Autonomy promotion domain:		
	Autonomy support	8.62	± 3.40
	Child involvement^a^	2.97	± 2.62
	Structure domain:		
	Meal routines	12.33	± 1.69
	Rules and limits	11.50	± 2.96
	Healthy opportunities	8.76	± 2.60
	Nondirective support	8.05	± 3.32

*N* = 315, with missing % representing the “*Prefer not to answer*” option. FPP values represent total score over the first 14-day period, ranging from 0 (never used) to 14 (used every day). ^a^Significant differences by parent sex.

**Fig. 1:**
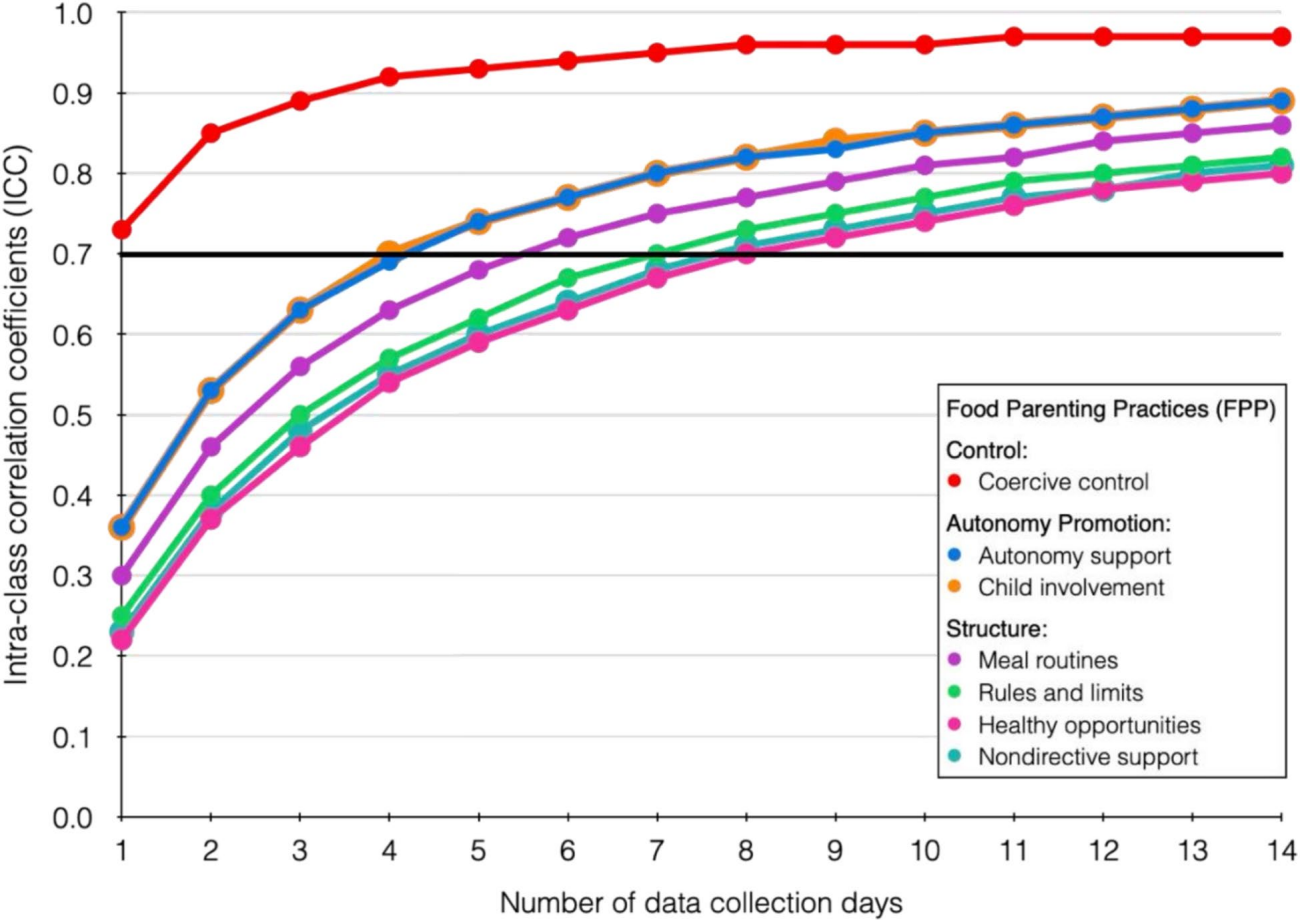
Reliability changes by number of data collection days (imputed data analysis, N = 315)

Table 2 presents ICC coefficients for the first 14-day assessment period, along with the minimum number of days required to achieve reliability ≥ 0.70. Figure 1 illustrates how reliability increased as the number of daily assessments accumulated. As expected, high reliability was achieved with 14 days of measurement. However, fewer days were sufficient for acceptable reliability. For three constructs (Autonomy support, Child involvement, and Meal routines), 5–6 days of data collection were enough to reach an ICC ≥ 0.70. Three constructs, Rules and limits, Healthy opportunities, and Nondirective support, required more than a week, while Coercive control showed high reliability with just one day. Table 2 also reports ICC values based on the second 14-day assessment period, comparing randomly selected consecutive and non-consecutive days using the previously estimated minimum number of days. For all FPP constructs, ICCs exceeded 0.70 when consecutive days were used. When the same number of days was selected non-consecutively, ICCs remained above 0.70 for all constructs except Nondirective support, which showed a borderline ICC = 0.68.

**Table 2:** Reliability and number of days needed for each FPP construct

	**First 14-day period**			**Second 14-day period**	
**FPP**	**ICC**_**i**_	**ICC**_**14**_	**Days needed**	**ICC**_**dc**_	**ICC**_**dr**_
Control domain:					
Coercive control	0.73	0.97	1	0.80	0.80
Autonomy promotion domain:					
Autonomy support	0.36	0.89	5	0.75	0.72
Child involvement	0.36	0.89	5	0.81	0.78
Structure domain:					
Meal routines	0.30	0.86	6	0.83	0.78
Rules and limits	0.25	0.82	8	0.92	0.91
Healthy opportunities	0.22	0.80	9	0.74	0.73
Nondirective support	0.23	0.81	8	0.72	0.68

*FPP* = Food parenting practices; ICC_i_ = ICC for a single day in the first assessment period; ICC_14_ = ICC over the first 14-day assessment period; ICC_dc_ = ICC in the second assessment period applying the estimated number of days (rounding up in all cases), selecting a random start day and including only consecutive days thereafter; ICC_dr_ = ICC in the second assessment period applying the estimated number of days, selecting random days from the second 14-day assessment period without the constrain of being consecutive. *N* = 315 (analyses conducted with imputed data)

**Fig. 2:**
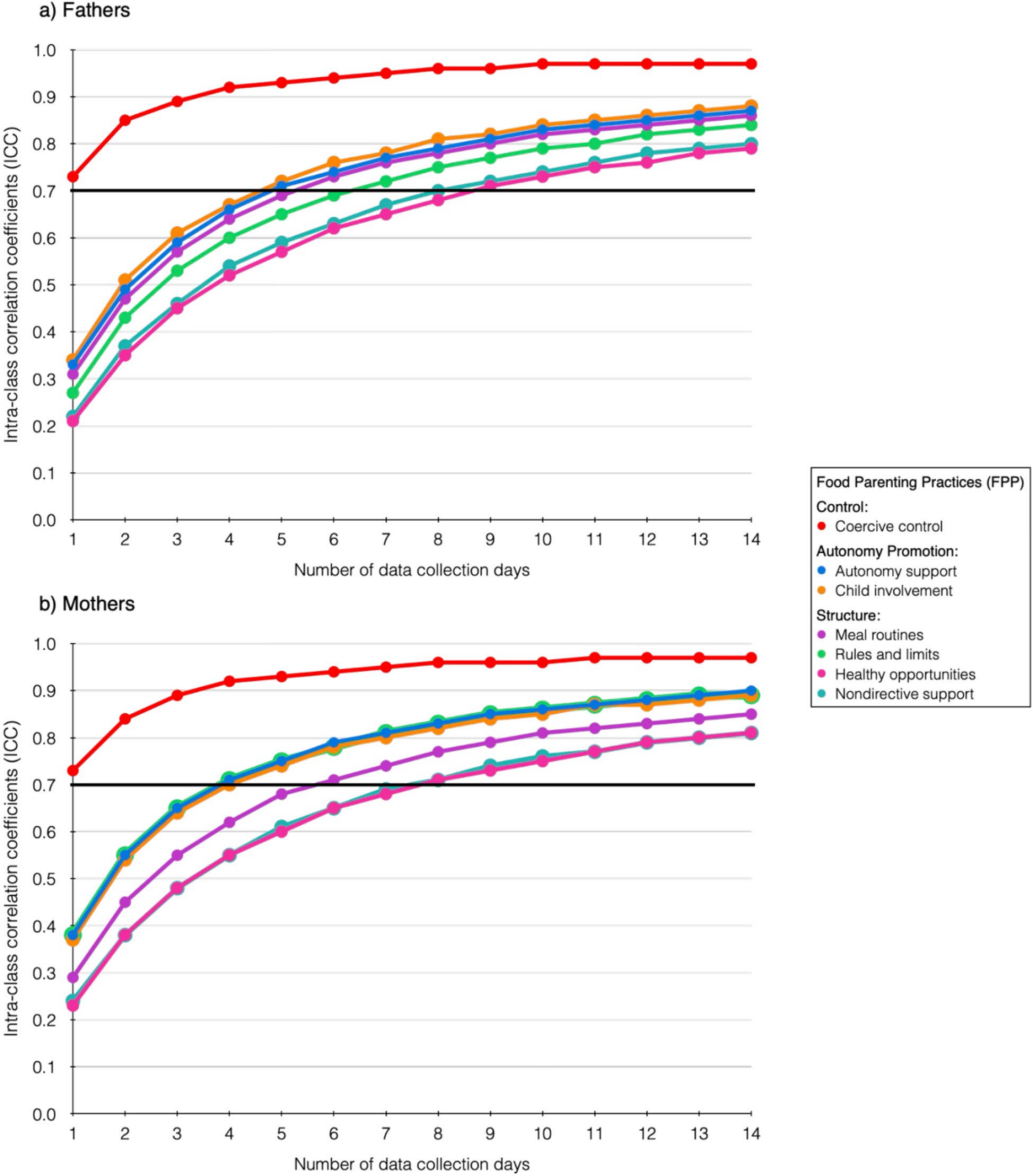
Reliability changes by number of data collection days (imputed data analyses, fathers *n* = 127, mothers *n* = 188)

Table 3 and Fig. 2 present the results stratified by parent sex. Despite the reduced sample size due to stratification, both fathers and mothers demonstrated adequate reliability in the first assessment period, with ICCs ranging from 0.79–0.97. Mothers required one fewer day than fathers to achieve reliable measurements for three constructs (Autonomy support, Healthy opportunities, and Nondirective support) and three fewer days for the reliable assessment of Rules and limits. In the second assessment period, reliability remained generally acceptable for both groups, with most ICC values exceeding the 0.70 threshold across caregiver types and day selection methods. However, a few borderline cases were observed. For fathers, Healthy opportunities had an ICC = 0.68 in the random day selection, while Nondirective support showed ICCs = 0.68 in both the consecutive and random day selections. Among mothers, Nondirective support showed an ICC = 0.68 with random day selection, while Autonomy support had ICCs = 0.68 and 0.63 in the consecutive and non-consecutive random day selection periods, respectively.

**Table 3:** Reliability and number of days needed for each FPP construct by parent sex

	**First 14-day period**			**Second 14-day period**	
	**ICC**_**i**_	**ICC**_**14**_	**Days needed**	**ICC**_**dc**_	**ICC**_**dr**_
Fathers’ FPP (*n* = 127)					
Control domain:					
Coercive control	0.73	0.97	1	0.84	0.84
Autonomy promotion domain:					
Autonomy support	0.33	0.87	5	0.77	0.71
Child involvement	0.34	0.88	5	0.82	0.78
Structure domain:					
Meal routines	0.31	0.86	6	0.85	0.79
Rules and limits	0.27	0.84	7	0.92	0.91
Healthy opportunities	0.21	0.79	9	0.74	0.68
Nondirective support	0.22	0.80	9	0.68	0.68
Mothers’ FPP (*n* = 188)					
Control domain:					
Coercive control	0.73	0.97	1	0.78	0.78
Autonomy promotion domain:					
Autonomy support	0.38	0.90	4	0.68	0.63
Child involvement	0.37	0.89	5	0.80	0.78
Structure domain:					
Meal routines	0.29	0.85	6	0.83	0.78
Rules and limits	0.38	0.89	4	0.81	0.81
Healthy opportunities	0.23	0.81	8	0.72	0.72
Nondirective support	0.24	0.81	8	0.75	0.68

*FPP* = Food parenting practices; ICC_i_ = ICC for a single day in the first assessment period; ICC_14_ = ICC over the first full 14-day assessment period; ICC_dc_ = ICC in the second assessment period applying the estimated number of days (rounded up in all cases), selecting a random start day and including only consecutive days thereafter; ICC_dr_ = ICC in the second assessment period applying the estimated number of days, selecting random days from the second 14-day assessment period without the constrain of being consecutive. N = 315 (analyses conducted with imputed data)

## Discussion

To our knowledge, our study is the first to use a Daily Diary design to examine the reliability of using simple binary items for repeated assessments of FPP among parents of 2.5–6-year-olds. Despite minor variations, our findings consistently showed that 14 days of data collection yielded high reliability across all FPP constructs, with most practices reaching acceptable reliability with as few as 5–6 days. Similar methodological approaches have been applied in other behavioral fields, most prominently movement behaviors (physical activity, sedentary time, and sleep), where device-based monitoring has largely been used to determine the minimum number of days needed for reliable estimates [30–32]. However, no prior work has extended these reliability methods to the intersection of nutrition and parenting. Our study advances this area by showing how reliability can be established in behaviors that are not directly captured by devices but instead must be self-reported within dynamic parent–child interactions that occur in the feeding context. This distinction is important because FPP reflect relational processes embedded within family routines and sociocultural contexts, differing meaningfully from individual-level behaviors objectively measured such as step counts or sleep duration.

More broadly, these findings suggest that relatively short diary protocols can yield reliable estimates of daily practices, reducing participant burden while maintaining data quality. This has implications not only for parenting and nutrition research but also for other relational and family-oriented domains of behavioral science that rely on diary or EMA methods, including studies of media use, physical activity parenting, sleep routines, and stress regulation within families. In applied contexts, these findings can guide survey design and implementation decisions. By identifying the minimum number of days needed for reliable assessment, researchers and practitioners can design protocols that minimize respondent burden and maximize engagement, particularly important in intervention studies where participant fatigue can compromise adherence. Shorter, targeted diary periods also allow more efficient use of research resources by reducing costs associated with data collection and processing. Together, these efficiencies may facilitate the integration of daily FPP assessments into behavior change interventions and program evaluations in real-world settings.

### Number of days needed for reliable assessments

The Spearman-Brown method [28] effectively estimated the number of days required to achieve reliable measurement across most FPP variables, which ranged from 1–9 days both in the full sample as well as in the sex-stratified analyses. Prior studies have relied on fixed assessment periods typically using a 5–10-day window and one or more assessments per day [2, 5–10, 13]. However, our findings demonstrate that a fixed period may be excessive for some FPP and insufficient for others. This has important implications for reducing participant burden and enabling researchers to allocate resources toward FPP constructs that require longer assessment periods.

Interestingly, across both the full sample and the sex-stratified analyses, Coercive control consistently required just one day for reliable assessment, whereas all other FPP constructs required about a week. Several factors may help explain this variability in reliability, particularly why controlling practices required significantly fewer assessment days. In our sample, Coercive control was reported on only about 10% of days (or roughly once during the 14-day period), yet still demonstrated high reliability. This suggests that parents who use this practice (~ 50% of the sample) do so consistently, while those who do not tend to avoid it consistently as well. These findings align with previous momentary research showing that controlling practices are used less frequently than structured or autonomy-promoting FPP [2, 5]. The high stability observed here may indicate that, compared to other FPP, controlling practices are more consistently applied when they do occur. Such practices may have a stronger dispositional basis [33], meaning their use (or absence) reflects a more ingrained and stable parenting tendency. When employed, they may function as habitual responses or coping mechanisms triggered by child behaviors, stressors, or events [5, 6, 12, 16]. Taken together, these findings suggest that shorter daily assessment periods may be sufficient to reliably capture controlling FPP, given their relatively high within-person stability.

The characteristics of our sample should be considered when interpreting these findings. Most participating families were well-resourced, highly educated, and in partnered households, which may help explain the relatively modest prevalence of controlling practices observed. Prior research grounded in the Family Stress Model [34] suggests that families experiencing greater socioeconomic stressors are more likely to rely on controlling strategies [34]. Therefore, our estimates may not fully reflect patterns present in more disadvantaged contexts. In addition, the meaning and function of controlling practices may vary across environments. For example, in families facing food insecurity, pressuring a child to eat may reflect concern that children consume available foods, rather than the same coercive dynamics observed in food-secure households [35, 36]. These contextual differences highlight how both the frequency and implications of controlling practices may differ depending on structural stressors. Future research should extend this work to socioeconomically diverse populations to evaluate whether the reliability patterns observed here generalize more broadly.

### Consecutive versus non-consecutive random day selection

In our study, the comparison between consecutive and non-consecutive day selection yielded similar ICC estimates, with high reliability in most cases. A subtle trend suggested that consecutive-day periods may produce slightly more reliable reports. Nevertheless, when consecutive assessments are not possible or are interrupted, incorporating additional non-consecutive days could help maintain acceptable reliability, which remained above 0.70 in most cases and above 0.60 in all. While not ideal, some studies have considered somewhat acceptable reliability when values are above 0.60, particularly in exploratory phases of research [25–27]. In Daily Diary studies, missing data is common, often due to participants failing to complete entire days rather than leaving individual items blank [14]. This can result in gaps in both predictors and outcomes for that day, making it difficult to maintain strict consecutive assessment periods. To our knowledge, most studies tend to prioritize consecutive-day periods and do not examine whether randomly selected days within a short window can still yield reliable estimates. Our findings suggest that including enough days, even if they are non-consecutive, can still yield reliable estimates, offering a flexible and pragmatic alternative for real-world data collection where daily compliance is imperfect.

Building on these results, researchers working with populations where lower compliance is expected can adapt protocols in several ways. Extending the overall data collection window allows participants to accumulate the minimum required number of days non-consecutively, thereby reducing burden while still ensuring acceptable reliability. Additionally, constructs that reached acceptable reliability with fewer days in our analyses could be prioritized when intensive protocols are not feasible. To further minimize participant burden, researchers could randomly assess different items each day, ensuring that each construct is evaluated for the required number of days without assessing all constructs daily. Researchers should also consider checking the percentage of missing data from similar studies to adjust the total number of days for reliable assessment. This ensures sufficient data even with missing values, and allows researchers to proactively follow up with participants or adjust their analysis accordingly. Finally, when compliance falls below ideal thresholds, reporting the achieved day count alongside corresponding reliability estimates provides transparency and allows readers to interpret findings in light of measurement precision. These strategies demonstrate how our findings can inform the design of diary-based assessments in more challenging research contexts.

### Fathers versus mothers

Finally, when comparing reliability estimates between fathers and mothers, some differences emerged. Both groups showed strong overall ICCs (0.79–0.97), but mothers required three fewer days to reliably assess Rules and limits, and one fewer day to reach acceptable reliability for Autonomy support, Healthy opportunities, and Nondirective support. This may reflect more consistent use of FPP among mothers, who have traditionally played a larger role in the feeding context [19, 37]. Interestingly, when applying the estimated number of days to the second assessment period, reliability generally remained above 0.70. However, borderline values were observed for Nondirective support in both parents, Healthy opportunities in fathers, and Autonomy support in mothers, with the latter dropping more notably when using non-consecutive days. Given that the average use of these practices did not differ significantly by parent sex, and that data collection was staggered across weekdays and seasons, this result is unlikely to reflect differences in frequency of use or timing of assessments, as reported in other studies [5, 11]. Instead, it may suggest that this behavior is embedded within structured, recurring feeding routines that unfold over multiple days (e.g., certain practices occurring every few days), making it harder to capture with randomly selected days. Alternatively, maternal Autonomy support may be highly context-dependent and therefore less consistently observed with non-continuous sampling. An EMA study [11] conducted predominantly with mothers, found that autonomy-supportive practices were more likely during mealtimes perceived as relaxed, enjoyable, or fun, and less likely when parents felt their child’s intake was “not enough”. These findings underscore the importance of aligning sampling strategies with the behavioral patterns being measured when designing intensive FPP assessments. Given the observed differences between parents, we recommend that future research tailor the assessment period based on parental sex and involvement in feeding routines. For mothers, a shorter assessment period may suffice for constructs like Rules and Limits, which are more consistently applied. For fathers, longer assessment periods should be considered to capture more variable feeding practices. Additionally, future studies should prioritize consecutive day sampling for constructs that are likely embedded in regular routines, as these may unfold over several days.

### Strengths and limitations

This study has several strengths stemming from its robust methodology and novel contributions. First, the longitudinal design, with two 14-day daily data collection periods, allowed for precise estimation of reliability, including the number of days needed to reach acceptable thresholds and the cross-validation of these estimates. Second, we examined a broad range of FPP constructs using a validated measure specifically developed for Daily Diary protocols, providing critical insight into both the frequency and stability of these practices, and offering practical guidance for future intensive assessments. Third, the once-daily design captured day-to-day fluctuations while minimizing recall bias, balancing data richness with participant burden. Fourth, by comparing reliability estimates based on consecutive versus randomly sampled days within a short window, this study offers valuable information for intensive longitudinal research, which is often challenged by missed or interrupted assessments. While non-consecutive days yielded comparable ICCs within a two-week period, future studies should examine whether this holds over longer durations. Fifth, the staggering of start dates and extended recruitment over ~ 15 months helped account for weekday and seasonal variability, increasing the ecological validity of our findings. Finally, the sample was balanced by parent and child sex and included ethnic diversity among parents, enhancing generalizability across these demographic characteristics.

However, several limitations should be considered. Generalizability is limited in terms of socioeconomic status and family structure, as the sample was predominantly composed of highly educated, higher-income, married or common-law two-parent families. In addition, reliance on self-reported data may introduce reporting and social desirability biases, particularly for parenting practices like Coercive control, which may be underreported due to social stigma [38]. This could influence estimates of how many days are needed to achieve reliable measurement, particularly if actual use is more frequent than reported. Finally, while the comparison of consecutive and non-consecutive day selections provides useful insights for studies where missing data may occur, these findings are most applicable to studies in which data are missing at random. In cases where missingness follows a systematic pattern, a different approach or new reliability assessments tailored to the observed missing data structure may be necessary.

## Conclusions

To our knowledge, this is the first study to examine the reliability of a broad range of FPP measures developed specifically for use in a Daily Diary protocol, and provides evidence on its internal structure via CFA. These findings contribute to a deeper understanding of the frequency and stability of FPP among mothers and fathers, while offering practical guidance on how to balance reliability, feasibility, and participant burden in intensive longitudinal studies. This study is also the first to compare reliability estimates based on consecutive versus randomly sampled days within a short timeframe, providing valuable insights for researchers facing missed or interrupted assessments, common challenges in Daily Diary research. While 14 days of data collection yielded high reliability across constructs, several FPP could be reliably measured with fewer days, allowing researchers to reduce participant burden without compromising data quality. Reliability estimates were comparable between consecutive and randomly selected days within a two-week window, supporting greater flexibility in scheduling. Although maternal and paternal FPP generally required a similar number of days to reach acceptable reliability, certain constructs appeared to fluctuate more depending on parent sex and measurement strategy. Future research should continue to evaluate the reliability of daily FPP assessments across more diverse populations.

## Supplementary Information

Supplementary Material 1: Table S1. Items used for daily assessments of food parenting practices (FPP) [39]. Table S2. Validity assessment of the instrument based on internal structure examined via Confirmatory Factor Analysis.

## Data Availability

The data used in this study are not publicly available, but can be made available on reasonable request. For data access requests, contact the corresponding author.

## Abbreviations

FPP: Food parenting practices
ICC: Intra-class correlation coefficients
GSM: Good Start Matters
RMSEA: Root mean squared error of approximation
CFI: Comparative fit index
TLI: Tucker-Lewis index
SRMR: Standardized root mean squared residual
