# Supplementary material for: The right dose of data: balancing reliability and participant burden in a validated daily measure of food parenting practices
Source: Int J Behav Nutr Phys Act. 2025 Nov 26;22:162. doi: 10.1186/s12966-025-01855-z (PMC12752353; doi:10.1186/s12966-025-01855-z)
Supplement: Supplementary file 1 — Supplementary Material 1: Table S1. Items used for daily assessments of food parenting practices (FPP) [39]. Table S2. Validity assessment of the instrument based on internal structure examined via Confirmatory Factor Analysis. [file 12966_2025_1855_MOESM1_ESM.docx]

# Supplemental Material

**Table S1**. Items used for daily assessments of food parenting practices (FPP)

| **FPP construct** | **Daily FPP assessments (current study)** | **FPP item bank** (21) |
| --- | --- | --- |
|  | *This set of questions will ask about your feeding practices with your child yesterday… (yes / no)* | *In the past month, how often did you… (never / rarely / sometimes / often / always)* |
| CONTROL | | |
| Coercive control | 1. Yesterday I took away dessert because [child name] misbehaved or did not eat all the food I served | Take away dessert as punishment for bad behavior |
|  | 1. Yesterday I made [child name] "clean the plate" to make sure my child ate enough food | Make your child stay at the table until all the food on his or her plate is eaten |
| AUTONOMY PROMOTION | | |
| Autonomy support | 1. Yesterday, I gave [child name] positive feedback (e.g., praised, gave a hug, a hand squeeze) for eating healthy foods | Say something nice to your child for tasting a NEW vegetable or food |
|  | 1. Yesterday, I talked about healthy foods and their importance in front of [child name] | Explain that eating healthy food will give your child more energy |
| Child involvement | 1. Yesterday, I involved [child name] in simple meal preparation tasks (e.g., washing the veggies, arranging food on the plate) | Have your child help prepare dinner meals & Have your child help you prepare vegetable dishes |
|  | 1. Yesterday, I asked [child name] to choose which vegetables to eat at dinner or for the snacks | Give your child a choice of vegetables to eat at dinner |
| STRUCTURE | | |
| Meal routines | 1. Yesterday we had dinner together as a whole family | Eat dinner together as a family (whole family) |
|  | 1. Yesterday I served [child name] their snacks and meals at the scheduled time (meaning at about the time when you expect your child to eat everyday) | N/A |
| Rules and limits | 1. Yesterday, [child name] did not like the food or snack I served, so I prepared something else to make sure my child ate something * | You expect your child to eat the foods that you serve or not eat at all |
|  | 1. Yesterday I limited [child name]’s intake of salty/sweet treats and sugary drinks (e.g., candies, chocolate, dessert, chips, pop, fruit drinks) | You limit how often your child eats/drinks sweet or salty treats (i.e., chips, desserts, sugary drinks) |
| Healthy opportunities | 1. Yesterday at dinner, I served a plate to [child name] where half of the plate had vegetables and fruit | N/A |
|  | 1. Yesterday at dinner, I served [child name] at least 2 different colorful vegetables (e.g., dark green, red, orange or purple vegetables) - DO NOT count potatoes or fried potatoes | Serve your child at least 2 different vegetables (excluding potatoes or fries) at dinner meals |
|  | 1. Yesterday at dinner, I served a plate to [child name] that included both a protein and a whole grain food? Only select "yes" if both conditions are met | N/A |
| Nondirective support | 1. Yesterday at dinner, I told [child name] how much I enjoyed the vegetables we ate | Show how much you enjoy eating vegetables while eating with your child |
| **Notes**: N/A indicates that there is not item in the item bank instrument available. In these cases, the daily FPP item was created for this study, in some cases to capture adherence to Canada’s Food Guide recommendations (39). * Item dropped post-validity assessment due to low factor loading. | | |

**Table S2**. Validity assessment based on internal structure examined via Confirmatory Factor Analysis

| **Overall Model Fit** | | |
| --- | --- | --- |
| Chi square test of model vs. saturated χ²(46)= 75.06, p=0.004 | | |
| Root Mean Squared Error of Approximation (RMSEA) =0.045 [0.25,0.63], 66% | | |
| Comparative Fit Index (CFI)=0.94 | | |
| Tucker-Lewis Index (TLI)=0.97 | | |
| Standardized Root Mean Squared Residual (SRMR)=0.04 | | |
| **Construct** | **Item** | **Factor loading (λ)** |
| Coercive control | 1. Yesterday I took away dessert because [child name] misbehaved or did not eat all the food I served | 0.57 |
|  | 1. Yesterday I made [child name] "clean the plate" to make sure my child ate enough food | 0.44 |
| Autonomy support | 1. Yesterday, I gave [child name] positive feedback (e.g., praised, gave a hug, a hand squeeze) for eating healthy foods | 0.75 |
|  | 1. Yesterday, I talked about healthy foods and their importance in front of [child name] | 0.85 |
| Child involvement | 1. Yesterday, I involved [child name] in simple meal preparation tasks (e.g., washing the veggies, arranging food on the plate) | 0.56 |
|  | 1. Yesterday, I asked [child name] to choose which vegetables to eat at dinner or for the snacks | 0.66 |
| Meal routines | 1. Yesterday we had dinner together as a whole family | 0.58 |
|  | 1. Yesterday I served [child name] their snacks and meals at the scheduled time (meaning at about the time when you expect your child to eat everyday) | 0.52 |
| Rules and limits | 1. Yesterday, [child name] did not like the food or snack I served, so I prepared something else to make sure my child ate something | Dropped * |
|  | 1. Yesterday I limited [child name]’s intake of salty/sweet treats and sugary drinks (e.g., candies, chocolate, dessert, chips, pop, fruit drinks) | 1.00 (restricted) |
| Healthy opportunities | 1. Yesterday at dinner, I served a plate to [child name] where half of the plate had vegetables and fruit | 0.82 |
|  | 1. Yesterday at dinner, I served [child name] at least 2 different colorful vegetables (e.g., dark green, red, orange or purple vegetables) - DO NOT count potatoes or fried potatoes | 0.76 |
|  | 1. Yesterday at dinner, I served a plate to [child name] that included both a protein and a whole grain food? Only select "yes" if both conditions are met | 0.36 |
| Nondirective support | 1. Yesterday at dinner, I told [child name] how much I enjoyed the vegetables we ate | 1.00 (restricted) |

Notes: * Item dropped post-validity assessment due t
